# Empiric antibiotic selection for community-acquired pneumonia in US hospitals, 2013–2020

**DOI:** 10.1017/ash.2023.249

**Published:** 2023-09-29

**Authors:** Hannah Wolford, Brandon Attell, James Baggs, Sujan Reddy, Sarah Kabbani, Melinda Neuhauser, Lauri Hicks

## Abstract

**Background:** Community-acquired pneumonia (CAP) is a common indication for antibiotic prescribing in hospitalized patients. Professional societies’ clinical guidelines recommend specific antibiotics for empiric treatment of CAP based on clinical factors. Manual assessments of appropriateness are time-consuming and are often conducted on a smaller scale. We evaluated empiric antibiotic selection among a large cohort of adults hospitalized with CAP using electronic health records. **Methods:** In this study, we used the PINC-AI healthcare database to define a cohort of adults hospitalized with CAP from 2013 to 2020. CAP was identified by *International Classification of Diseases* (ICD) diagnosis codes. Exclusions were applied to identify uncomplicated CAP (Fig. 1). Treatment was only evaluated if a chest radiograph or computerized tomography (CT) scan was charged during the first 2 days of hospitalization, otherwise it was considered an inadequate CAP evaluation. Administrative billing data were used to identify antibiotics charged within the first 2 days of hospitalization. Empiric guideline-recommended treatment was determined based on 2019 CAP guidelines and more recent studies. Patients who received nonrecommended treatment were evaluated for antibiotic allergies in the current hospitalization or methicillin-resistant *Staphylococcus aureus* (MRSA) colonization or infection in the year prior or on admission using *International Classification of Disease, Tenth Revision* (ICD-10) diagnosis codes. **Results:** We identified 4.47 million adult hospitalizations with CAP from 2013 to 2020; 32% (1.43 million) were included in this analysis (Fig. 1). Among discharges with adequate CAP evaluation (1.37 million), 59.7% received recommended antibiotics in the first 2 days of hospitalization, ranging from 62.6% in 2013 to 57.5% in 2019. Overall, 34.8% of our study population received a nonrecommended antibiotic without documentation of an antibiotic allergy or MRSA colonization (2013: 32.5%; 2018: 36.7%) (Fig. 2). Most patients in our study population received >1 antibiotic (92.3%) in the first 2 days of hospitalization. The most common antibiotics among patients receiving recommended treatment were ceftriaxone (74.2% of patients receiving recommended treatment), azithromycin (67.2%), and levofloxacin (31.8%) (Fig. 3a). The most common nonrecommended antibiotics were vancomycin (57.2% of patients receiving nonrecommended treatment), piperacillin-tazobactam (48.1%), and cefepime (25.7%) (Fig. 3b). From 2013 to 2020, cefepime charges consistently increased among CAP patients treated with nonrecommended antibiotics, whereas levofloxacin charges consistently decreased among CAP patients treated with only recommended antibiotics. **Conclusions:** Approximately one-third of patients with uncomplicated CAP received nonrecommended empiric antibiotics, and from 2013 to 2020 that proportion increased by 9%. Additional strategies are needed to help identify opportunities to optimize antibiotic selection among patients with CAP.

**Figure:**
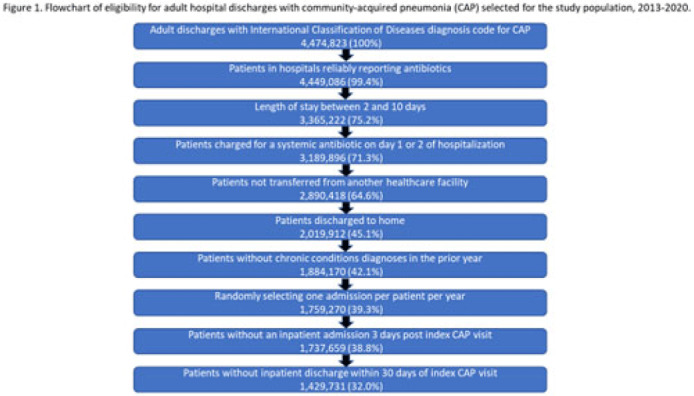

**Figure:**
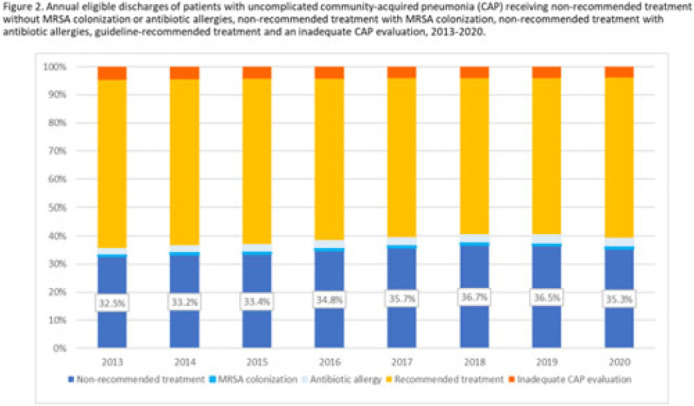

**Figure:**
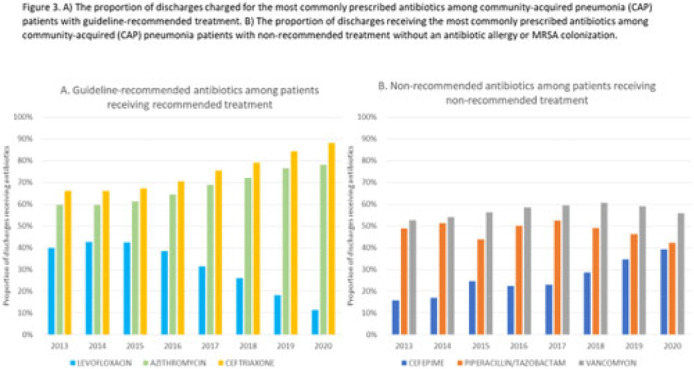

**Disclosures:** None

